# Case report and literature review: endoscopic extended endonasal resection of two cases of giant chondroma

**DOI:** 10.3389/fsurg.2025.1575229

**Published:** 2025-05-16

**Authors:** Cao Yingxiao, Wang Hao, Shang Shuling, Zhang Xike, Feng Qingye

**Affiliations:** Department of Neurosurgery, Xingtai People’s Hospital Affiliated to Hebei Medical University, Xingtai, China

**Keywords:** intracranial chondromas, skull base tumor, neuroendoscope, cerebral tumor, neurosurgery

## Abstract

**Background:**

Intracranial chondromas are exceedingly rare neoplasms, accounting for 0.2% to 0.3% of all intracranial tumors. These tumors predominantly originate from the skull base, particularly at the cartilaginous junctions of the cranial sutures, with the sella turcica being the most common site.

**Case presentation:**

Between April 2022 and August 2023, the Department of Neurosurgery at Xingtai People’s Hospital Affiliated to Hebei Medical University treated two cases of giant skull base chondromas using endoscopic extended endonasal resection. Near-total resection was achieved, and postoperative histopathological examination confirmed the diagnosis of chondroma. Both patients recovered well postoperatively.

**Conclusion:**

Skull base chondromas are deeply seated and often involve extensive regions. Complete surgical resection remains the greatest challenge and the most critical factor influencing prognosis.selecting the appropriate surgical approach and achieving complete endoscopic resection can effectively prevent tumor recurrence and improve patient quality of life.

## 1 Introduction

Chondromas are benign tumors arising from hyaline cartilage. The age of onset typically ranges from 20 to 60 years, with a peak incidence between 20 and 30 years and a mean age of 29 years. There is no significant gender predilection, although some studies suggest a slight female predominance (1). Chondromas can occur in any part of the body, but intracranial chondromas are exceptionally rare, constituting only 0.2% to 0.3% of intracranial tumors (2). These tumors most commonly originate from the skull base, particularly at the cartilaginous junctions of the cranial sutures, such as the sella turcica. Other potential sites of origin include the tentorium cerebelli, falx cerebri, brain parenchyma, leptomeninges, and choroid plexus (3). Although intracranial chondromas are generally considered benign, they may occasionally be associated with syndromes such as Ollier's disease (multiple enchondromatosis) or Maffucci syndrome.

Since 2022, our institution has treated two cases of intracranial chondroma confirmed by endoscopic extended endonasal surgery and histopathology. Below, we present the clinical characteristics, literature review, and therapeutic insights from these cases.

## 2 Materials and methods

### 2.1 Case 1

A 55-year-old female presented with a 5-year history of dizziness and vomiting without an identifiable cause, leading to the discovery of an intracranial tumor. She was initially treated at a hospital in Hebei Province, where she underwent a right subtemporal approach under general anesthesia on May 22, 2017, for the resection of a right middle and posterior cranial fossa lesion extending across the petrous apex. Postoperative pathology suggested a chondroma. Postoperatively, she developed right-sided facial hypoesthesia, right-sided masticatory weakness, and limited abduction of the right eye. Physical examination revealed limited abduction of the right eye, blurred vision when looking to the right, right-sided facial hypoesthesia, right-sided masticatory muscle weakness, left ear hearing loss, left limb muscle strength of grade 4, normal muscle strength in both upper limbs and the right lower limb, and a positive Romberg's sign.

**Figure 1:**
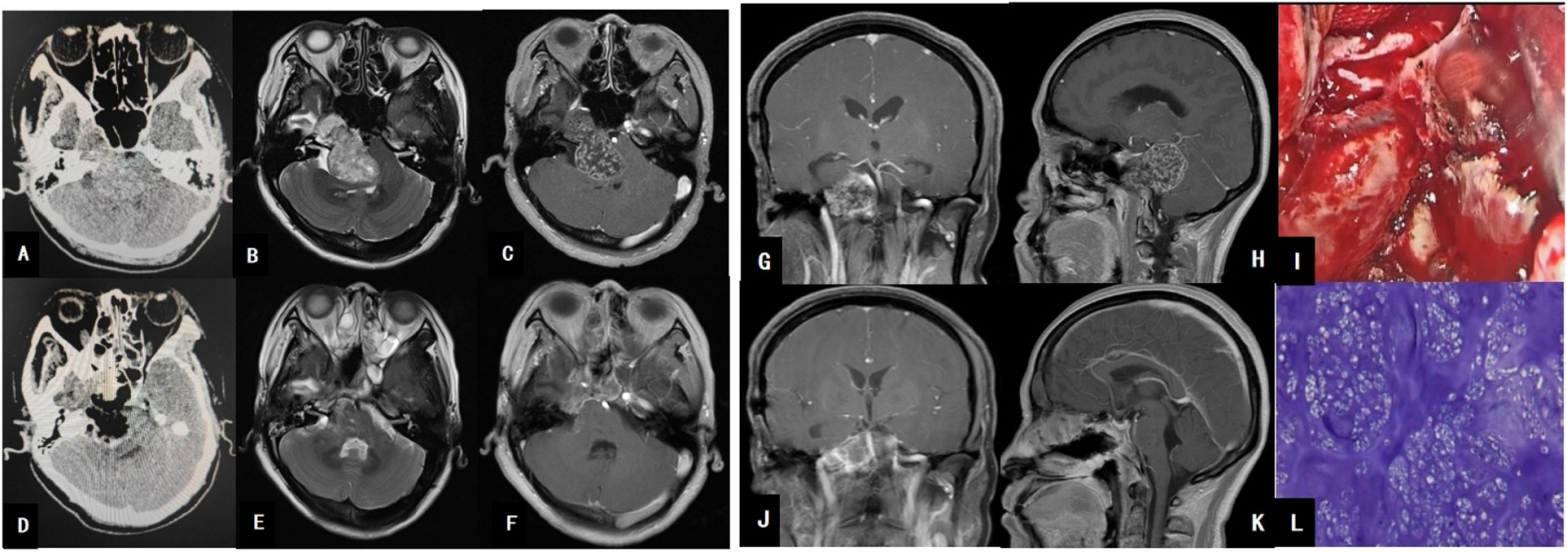
**(A)** Brain CT shows a large hyperdense mass in the right middle and posterior cranial fossa. **(B)** MRI T2 reveals a large hyperintense mass in the middle and posterior cranial fossa. **(C)** MRI T1 axial contrast-enhanced image demonstrates heterogeneous enhancement of the large tumor, closely related to the petrous segment of the internal carotid artery. **(G)** MRI T1 coronal contrast-enhanced image shows the tumor’s close relationship with the basilar artery. **(H)** MRI T1 sagittal contrast-enhanced image reveals tumor compression on the cerebellum and brainstem. **(I)** Intraoperative view of tumor resection. **(D)** Postoperative CT changes after tumor resection. **(E,F,J,K)** Postoperative MRI changes, indicating complete tumor resection. **(L)** Pathological image of the tumor.

Head CT imaging demonstrated a large mixed-density lesion in the right middle and posterior cranial fossa, containing multiple calcified spots and low-density areas, with an irregular, broad, high-density band at the lesion margin. Head MRI revealed a dumbbell-shaped soft tissue mass in the right cerebellopontine angle, extending across the middle and posterior cranial fossa, exhibiting slightly long T1 and T2 signals, with small patchy areas of signal reduction and heterogeneous enhancement. The tumor measured approximately 5.3 cm × 2.9 cm × 3.7 cm, displacing the right internal carotid artery laterally and compressing the adjacent brainstem and fourth ventricle (see Figure 1).

Using neuroendoscopy for close observation, the surgical field was well-visualized, with extensive exposure and ample operating space. A two-surgeon, four-handed technique was employed, with the assistant holding the endoscope and suction device while the primary surgeon operated with both hands. A modified Denker's approach and transpterygoid approach were utilized, beginning with endoscopic resection of the right middle turbinate, followed by drilling open the anterior and medial walls of the right maxillary sinus, severing the right nasolacrimal duct, resecting the right ethmoid bulla, opening the right ethmoid sinus, and drilling the anterior and lateral walls of the sphenoid sinus to expose the tumor. The maxillary nerve was displaced laterally by the tumor, which was located in the right sphenopetroclival region. The tumor was firm, white, lobulated, and partially calcified, with moderate blood supply, and displaced the right internal carotid artery laterally. The tumor was resected piecemeal using pathological forceps, with deep resection extending to the posterior cranial fossa, where the basilar artery was visualized. The tumor boundaries were clear, and gross total resection was achieved. A defect in the posterior fossa dura was repaired using artificial dura and a mucosal flap.

Postoperative pathology revealed focal areas of nuclear atypia, with immunohistochemistry results showing CD99 (+), S-100 (+), and Ki-67 (approximately +1%), consistent with a chondroma. Postoperatively, the patient developed a low-flow cerebrospinal fluid (CSF) leak, which was identified on neuroendoscopic examination as a small defect in the sellar floor. The leak was resolved by packing the mucosal flap with iodoform gauze, and the patient was discharged (see Figure 1).

At the 3-year follow-up, the patient's left limb muscle strength had returned to normal, but there was no significant improvement in the limited abduction of the right eye, blurred vision when looking to the right, right-sided facial hypoesthesia, right-sided masticatory weakness, or left ear hearing loss. The Karnofsky Performance Status (KPS) score was 80. Follow-up MRI scans at 6 months, 1 year, 2 years, and 3 years postoperatively showed no evidence of tumor recurrence.

### 2.2 Case 2

**Figure 2:**
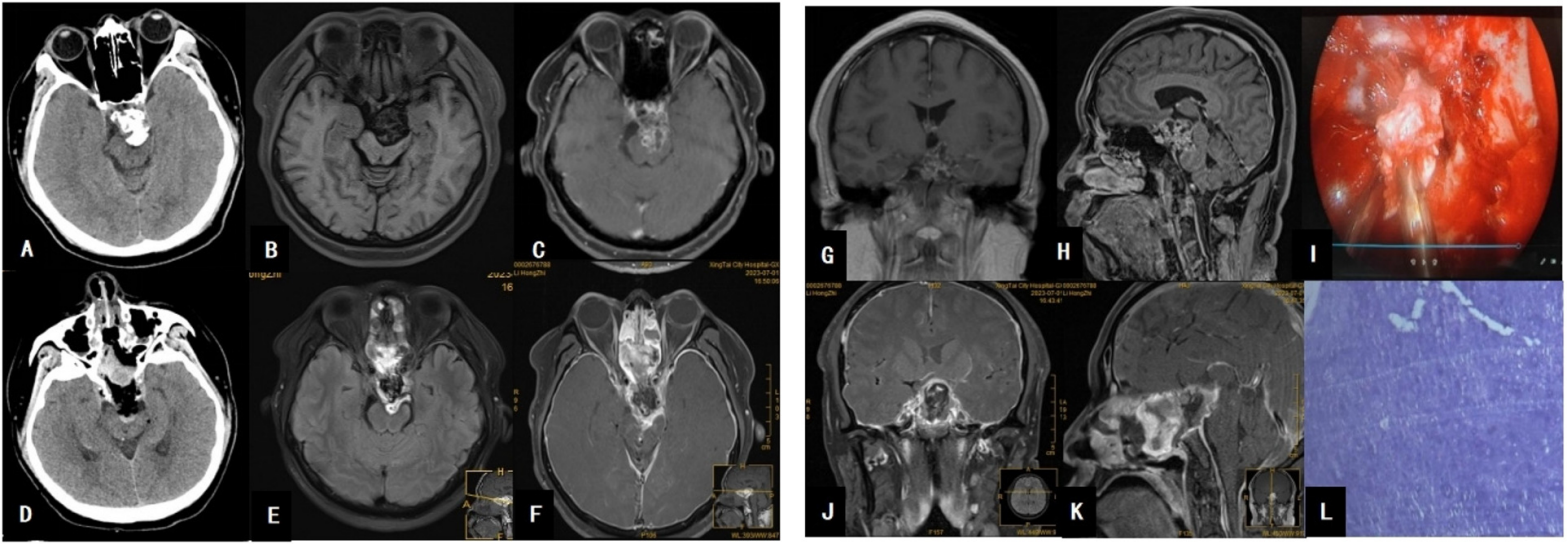
**(A)** Brain CT shows a hyperdense mass posterior to the sella turcica. **(B)** MRI T1-weighted image reveals a hypointense signal posterior to the sella turcica and anterior to the brainstem. **(C)** MRI T1 axial contrast-enhanced image demonstrates heterogeneous enhancement of the large tumor, which encases the basilar artery. **(G)** MRI T1 coronal contrast-enhanced image shows the tumor’s close relationship with the left cavernous sinus. **(H)** MRI T1 sagittal contrast-enhanced image reveals tumor compression on the brainstem, resulting in brainstem deformation. **(I)** Intraoperative view of tumor resection; the tumor exhibits a firm texture. **(D)** Postoperative CT changes after tumor resection. **(E,F,J,K)** Postoperative MRI changes, indicating complete tumor resection. **(L)** Pathological image of the tumor.

A 40-year-old male presented with a 12-day history of blurred vision without an identifiable cause. Physical examination revealed left eye visual acuity of 0.2, left ptosis, and limited movement of the left eyeball, with normal fundus findings. CT imaging demonstrated an irregular high-density lesion in the left dorsum sellae and interpeduncular fossa, predominantly composed of bony density and calcifications, interspersed with soft tissue density. MRI revealed slightly long T1 and T2 signals in the left dorsum sellae and interpeduncular fossa, with small patchy areas of signal reduction and heterogeneous enhancement. The tumor, measuring approximately 4.6 cm × 2.7 cm × 3.3 cm, was closely associated with the midbrain and compressed the basilar artery, raising suspicion for either a chordoma or chondroma (see Figure 2).

During surgery, the right superior turbinate was endoscopically resected, and the sellar floor was extensively drilled. The left cavernous sinus dura was incised, and the left pituitary gland was displaced. The inferior hypophyseal artery was coagulated, and the pituitary trabeculae were cut. The sellar floor dura was incised, and the dorsum sellae was drilled to expose the tumor, which was firm, white, lobulated, and partially calcified, with moderate blood supply. The tumor, rich in calcifications, was completely resected using pathological forceps. The sellar floor was reconstructed using artificial dura, fat, and a nasal septal mucosal flap.

Postoperative pathology revealed abundant cartilage tissue, fragmented bone tissue, and bone marrow cavities containing hematopoietic cells. Immunohistochemistry results were as follows: AE1/AE3 (−), Vimentin (+), S-100 (+), EMA (−), GFAP (−), Brachyury (−), SMA (−), P53 (wild-type), E-cad (−), B-catenin (−), and Ki-67 (approximately 5%). The findings were consistent with an osteochondroma (see Figure 2).

Postoperatively, the patient developed a fever, and lumbar puncture indicated intracranial infection, which was managed with antibiotics and lumbar cistern drainage. The infection was controlled, and the patient was discharged.

At the 2-year follow-up, the patient's left eye visual acuity improved to 0.4, with partial resolution of left ptosis but persistent limited movement of the left eyeball. The Karnofsky Performance Status (KPS) score was 90. Follow-up MRI scans at 6 months, 1 year, and 2 years postoperatively showed no evidence of tumor recurrence.

## 3 Discussion

Intracranial chondromas are exceedingly rare, accounting for approximately 0.06% of primary intracranial tumors. Leithof reported 4 cases of chondroma among 4,135 brain tumors (1), while Kleinsasser and Friedmann identified 9 cases among 6,000 intracranial tumors. Solitary intracranial chondromas typically originate from the dura, choroid plexus, leptomeninges, or brain parenchyma at the skull base (2). The skull base develops through endochondral ossification, which may explain the presence of residual chondrocytes and the predilection for chondromas in this region. Most tumors are extradural, as seen in our cases.

The age of onset for intracranial chondromas ranges from 20 to 60 years, with a peak incidence around 30 years. However, cases as young as 15 months have been reported (3). There is no significant gender predilection, although some studies suggest a slight female predominance (4). Clinical manifestations result from local compression of surrounding structures and may include symptoms of increased intracranial pressure due to cerebrospinal fluid pathway obstruction (1). Due to their nonspecific presentation, diagnosis is challenging.

Neuroimaging plays a crucial role in localization, but definitive diagnosis can be difficult, particularly in distinguishing chondromas from chordomas. Preoperative misdiagnosis is common. Imagawa (5) reported that intracranial chondromas are often confused with chordomas, skull base meningiomas, epidermoid cysts, and craniopharyngiomas. This is due to their rarity and overlapping clinical and radiological features with other skull base tumors. Key radiological features include destruction of normal skull base structures, calcifications, and ossifications on x-ray. CT scans reveal high-density, heterogeneous lesions with calcifications, while MRI shows mixed signals, with T2-weighted images demonstrating high-to-intermediate signal intensity and low signal intensity in calcified areas (6).

Intracranial chondromas, as demonstrated in the two cases above, exhibit distinct pathological characteristics. Grossly, the tumors are firm, pearl-like, and white, with a lobulated and irregular surface often covered by a thin, translucent capsule. Histopathological examination reveals multiple calcified foci, with the tumor composed of lobules of cartilaginous tissue rich in hyaline matrix. Some chondrocytes within the tumor display abundant cytoplasm and hyperchromatic nuclei, with minimal intercellular spaces between adjacent chondrocytes. The tumor matrix contains islands of both newly formed, active woven bone and older bone with Haversian systems. Staining of the tumor matrix reveals the presence of acid mucopolysaccharides. Notably, histological sections of the tumor-bearing cranial bone show no evidence of tumor invasion, indicating that the tumor does not infiltrate the surrounding osseous structures (7).

The treatment of intracranial chondromas primarily involves surgical resection as the main therapeutic approach; however, due to the tumors' frequent location at the skull base and their expansive growth pattern, they often reach a considerable size before manifesting symptoms and clinical signs. Additionally, their proximity to critical structures such as the brainstem, cranial nerves, and major blood vessels at the skull base complicates surgical intervention. Historically, surgical approaches such as the subtemporal, extended pterional, far-lateral, and transsphenoidal routes under microscopic guidance have been employed, but these methods are associated with limited exposure, significant trauma, restricted operative fields, and substantial challenges and risks in achieving complete tumor resection, resulting in low rates of gross total removal. Postoperative stereotactic radiotherapy, while commonly administered, has shown limited efficacy in inhibiting tumor cell proliferation, is associated with significant radiation-induced damage, and is linked to a high rate of tumor recurrence.

With the advancement of endoscopic techniques and improvements in skull base reconstruction, the use of neuroendoscopy, leveraging its superior illumination and magnification, allows for clearer visualization of the lesion site, enhancing surgical precision and minimizing damage to surrounding normal tissues. Additionally, the flexibility to tailor the surgical approach and extent of resection based on the specific clinical condition and anatomical structure enables better preservation of the physiological functions of the nasal cavity and paranasal sinuses, thereby reducing the incidence of postoperative complications. Compared to traditional craniotomy, microscopic transcranial surgery requires scalp incision and craniotomy, which is associated with significant trauma. Postoperatively, patients often experience pronounced head wound pain, potentially necessitating strong analgesic medications for relief, and the wound healing process is prolonged, with pain persisting for an extended period. Recovery is slow, requiring prolonged bed rest and close monitoring of vital signs to prevent complications such as intracranial hemorrhage or infection. Patients may also experience headaches, dizziness, nausea, and other discomforts, with physical recovery taking weeks or even months. The procedure leaves a noticeable surgical scar on the head, which can significantly impact appearance, particularly in patients prone to keloid formation, potentially affecting their psychological well-being. Furthermore, microscopic transcranial surgery may lead to neurological dysfunction due to intraoperative brain tissue manipulation, manifesting as memory decline, speech difficulties, or motor impairments. Risks such as intracranial infection, cerebral edema, and associated symptoms like fever, headache, and vomiting also remain significant concerns.

In endoscopic endonasal surgery, the procedure is performed through the natural nasal passage, leaving no external incisions and causing minimal trauma to the nasal cavity. Utilizing a two-surgeon, four-handed technique, the assistant holds the endoscope and suction device while the primary surgeon operates with both hands, allowing for close observation and a larger operative space. For the two cases of large skull base chondromas, as demonstrated by the cranial CT and MRI scans in Case 1 and Case 2, modified Denker's approach, transpterygoid approach, and pituitary transposition techniques were employed to expand the exposure, effectively transforming the skull base tumor into a convex lesion, enabling complete tumor resection. Postoperative pain is relatively mild, typically presenting as slight nasal distension or discomfort, which can generally be alleviated with routine analgesic measures. Recovery is faster, with minimal impact on daily life. In the absence of complications, patients can ambulate shortly after surgery, and physical function recovers quickly, often allowing a return to normal activities within approximately one week. However, there may be minor bloody nasal discharge, and some time is required for the restoration of normal nasal airflow.

Endoscopic endonasal surgery, being an internal nasal procedure, leaves no visible external scars, thus avoiding any impact on facial appearance and reducing psychological distress related to cosmetic concerns for patients. Potential side effects of endoscopic endonasal surgery include dry nasal mucosa, minor bleeding, and transient hyposmia. Some patients may experience nasal soreness, rhinorrhea, or similar discomforts, which can generally be managed with symptomatic treatment. Severe complications are relatively rare.

The extent of surgical resection is a critical factor influencing the recurrence rate of tumors. Achieving gross total resection can significantly reduce the likelihood of recurrence; however, the intricate anatomy of the skull base often makes complete resection challenging. In some small retrospective studies, the recurrence rate following subtotal resection has been reported to be as high as 70%, whereas gross total resection can lower the recurrence rate to approximately 30%. The location of the tumor also plays a significant role in recurrence. For instance, chondromas located in regions such as the clivus or petrous apex, where critical structures are densely concentrated and surgical maneuverability is limited, are more difficult to completely remove, resulting in a higher risk of recurrence. Compared to chondromas in relatively isolated areas of the anterior skull base, tumors in these complex regions exhibit a recurrence rate that is 2 to 3 times higher (8).

In addition, the pathological subtype also influences the recurrence rate. Classic chondromas have a relatively lower recurrence rate, whereas mesenchymal chondromas, due to their more aggressive biological behavior, exhibit a significantly higher recurrence rate, ranging from 50% to 80%. A multicenter study revealed that the time to recurrence in patients with mesenchymal chondromas is notably shorter than in those with classic chondromas, with average recurrence times of 3 years and 5 years, respectively (9).

Despite continuous advancements in surgical techniques, including the use of adjuncts such as neuronavigation and intraoperative neurophysiological monitoring, which have improved the safety and thoroughness of surgical resection to some extent, the issue of postoperative recurrence in skull base chondromas remains unresolved. Recurrent skull base chondromas pose greater surgical challenges and carry a higher risk of neurological damage during reoperation.

With the ongoing development of radiotherapy, Tomotherapy offers highly conformal and high-dose radiation treatment, enabling more precise tumor targeting and better protection of surrounding normal tissues compared to conventional radiotherapy. Proton therapy for skull base chondromas has demonstrated a 5-year local control rate of 78% and a 5-year survival rate of 85%. Research from Massachusetts General Hospital in the United States shows that pediatric and adolescent patients receiving proton beam radiation therapy have 5-year, 10-year, and 20-year overall survival rates of 96.2%, 93.3%, and 89.4%, respectively (9). These advanced radiotherapy techniques offer several benefits: due to their unique physical properties, proton therapy can precisely target tumors while minimizing radiation exposure to surrounding normal tissues, thereby reducing radiotherapy-related toxicities and lowering the risk of recurrence. For patients who have undergone surgery for skull base chondromas, these techniques help eradicate residual tumor cells, reduce the risk of local recurrence and distant metastasis, and improve overall survival rates.

Therefore, endoscopic endonasal surgery enables either complete or near-total resection of the tumor, with the primary goal of relieving compression on critical structures such as the brainstem. In cases where gross total resection is achieved, postoperative radiotherapy may not be necessary. However, for subtotal resection, adjuvant radiotherapy is typically required to suppress tumor recurrence and improve long-term outcomes.

We believe that skull base chondromas, due to their deep anatomical location and extensive involvement, present significant challenges in achieving complete surgical resection, which is the most critical factor influencing prognosis. The adequacy of surgical exposure directly impacts the extent and degree of tumor resection. Traditional microscopic transnasal surgery often faces limitations in exposure, whereas expanded endoscopic endonasal approaches provide sufficient surgical visibility and are well-suited for skull base chondroma resection. During the procedure, it is essential to protect critical structures such as the internal carotid artery, optic nerve, oculomotor nerve, and skull base anatomy, while also preventing cerebrospinal fluid leakage. Maximizing the use of natural sinus cavities and working gently within these spaces are crucial. Careful identification of the internal carotid artery and cranial nerves, along with timely irrigation of the surgical field with papaverine, helps prevent spasms of the internal carotid artery and its small perforating branches. Postoperatively, the skull base should be reconstructed using absorbable artificial dura, pedicled mucosal flaps, and a “sandwich” technique with biological glue. If necessary, fascia lata and fat grafts can be added to reinforce the repair. In summary, selecting an appropriate surgical approach and achieving complete tumor resection under endoscopy can effectively prevent tumor recurrence and significantly improve the patient's quality of life.

## Data Availability

The original contributions presented in the study are included in the article/Supplementary Material, further inquiries can be directed to the corresponding author.
